# Targeting RPA promotes autophagic flux and the antitumor response to radiation in nasopharyngeal carcinoma

**DOI:** 10.1186/s12967-023-04574-w

**Published:** 2023-10-19

**Authors:** Yanchun Feng, Yingming Jiang, Jun Liu, Jiaqi Liu, Mengchen Shi, Junxiong Chen, Jingdan Zhang, Yu Tian, Xiangling Yang, Huanliang Liu

**Affiliations:** 1 Department of Clinical Laboratory, The Sixth Affiliated Hospital, Sun Yat-Sen University, Guangzhou, 510655 China; 2 Guangdong Institute of Gastroenterology, Guangzhou, 510655 China; 3 Department of General Surgery, The Sixth Affiliated Hospital, Sun Yat-Sen University, Guangzhou, 510655 China; 4 Guangdong Provincial Key Laboratory of Colorectal and Pelvic Floor Diseases, The Sixth Affiliated Hospital, Sun Yat-Sen University, Guangzhou, 510655 China; 5 Biomedical Innovation Center, The Sixth Affiliated Hospital, Sun Yat-Sen University, Guangzhou, 510655 China; 6 Department of General Surgery (Department of Gastric Surgery Section 2, ), The Sixth Affiliated Hospital, Sun Yat-Sen University, Guangzhou, 510655 China

**Keywords:** RPA, Nasopharyngeal carcinoma, Autophagy, Radiotherapy

## Abstract

**Background:**

Autophagy is involved in nasopharyngeal carcinoma (NPC) radioresistance. Replication protein A 1 (RPA1) and RPA3, substrates of the RPA complex, are potential therapeutic targets for reversing NPC radioresistance. Nevertheless, the role of RPA in autophagy is not adequately understood. This investigation was performed to reveal the cytotoxic mechanism of a pharmacologic RPA inhibitor (RPAi) in NPC cells and the underlying mechanism by which RPAi-mediated autophagy regulates NPC radiosensitivity.

**Methods and results:**

We characterized a potent RPAi (HAMNO) that was substantially correlated with radiosensitivity enhancement and proliferative inhibition of in vivo and in NPC cell lines in vitro. We show that the RPAi induced autophagy at multiple levels by inducing autophagic flux, AMPK/mTOR pathway activation, and autophagy-related gene transcription by decreasing glycolytic function. We hypothesized that RPA inhibition impaired glycolysis and increased NPC dependence on autophagy. We further demonstrated that combining autophagy inhibition with chloroquine (CQ) treatment or genetic inhibition of the autophagy regulator ATG5 and RPAi treatment was more effective than either approach alone in enhancing the antitumor response of NPC to radiation.

**Conclusions:**

Our study suggests that HAMNO is a potent RPAi that enhances radiosensitivity and induces autophagy in NPC cell lines by decreasing glycolytic function and activating autophagy-related genes. We suggest a novel treatment strategy in which pharmacological inhibitors that simultaneously disrupt RPA and autophagic processes improve NPC responsiveness to radiation.

**Supplementary Information:**

The online version contains supplementary material available at 10.1186/s12967-023-04574-w.

## Background

Nasopharyngeal carcinoma (NPC) is an aggressive form of cancer that lacks differentiation. Due to the radiosensitivity and anatomic location of NPC, radiotherapy is the standard treatment [1]. While radiotherapy is currently regarded as the most effective treatment for NPC, the emergence of radiation resistance and tumor recurrence following irradiation are major hindrances to successful treatment [2]. To improve the prognosis for NPC patients and increase 5-year survival rates, researchers have focused on preventing radioresistance and enhancing radiosensitivity [3]. Furthermore, the underlying mechanisms that lead to the growth and metastasis of NPC need to be fully clarified, highlighting the need for effective therapeutic strategies for the treatment of patients with NPC.

Radiation-induced cell death occurs through the induction of deoxyribonucleic acid (DNA) damage, which may then lead to apoptosis, senescence and autophagy [4]. Autophagy is primarily described as a mechanism of radioresistance in cancer, but its exact role is complex and unclear, which has impeded progress in the development of agents targeting autophagy for cancer treatment [5]. Autophagy, the lysosomal breakdown of cytosolic components, is a crucial homeostatic process that is triggered when subjected to stress and appears to be related to DNA damage [6]. This process is critical for the maintenance and structural reconstruction of cell homeostasis by eliminating aging cells or those with internal organ damage, misfolded proteins, and invading pathogens. The formation of autophagosomes is the morphological hallmark of autophagy and requires the involvement of multiple factors, including Beclin1, LC3B, and p62 [7]. Several signaling molecules restrain this process, with the adenosine 5′-monophosphate (AMP)-activated protein kinase (AMPK)/mammalian target of rapamycin (mTOR) signaling pathway being the definitive pathway implicated in autophagic control [8].

The DNA-binding protein RPA (RPA1, 2, and 3) is a heterotrimeric protein that functions in the ataxia telangiectasia mutated protein (ATM)/ATR-mediated DNA damage response (DDR) [9]. Moreover, (1Z)-1-[(2-hydroxyanilino) methylidene] naphthalen-2-one (HAMNO) is a new protein interaction inhibitor of RPA that specifically blocks RPA1′s interaction with ATR, and thus, checkpoint engagement in the context of replication stress is suppressed [10, 11]. Exome-wide association analysis in our earlier investigation revealed RPA1 to be a unique predictive biomarker for NPC. Evidence from both gain- and loss-of-function experiments indicated that RPA1 aided in the development, invasion, metastasis, and radioresistance of NPC cells. Additionally, our lab suggested that increasing radiation sensitivity in NPC by therapeutically targeting RPA1 may be possible, but the mechanism behind this effect remains unknown [12].

Here, we demonstrated that pharmacological suppression of RPA alone in the current research inhibited proliferation via apoptotic mechanisms and increased radiosensitivity in NPC cells. Mechanistically, the improved effectiveness of concurrent RPA and radiation and/or autophagy inhibition with chloroquine (CQ) may be explained by the fact that RPA inhibition activated AMPK, which induced autophagy. Overall, our findings indicate that a treatment approach including inhibitor combinations that concurrently block various metabolic pathways, including autophagy, may be extremely successful for enhancing NPC sensitivity to radiation.

## Methods

### Cell culture

SYSUCC provided human NPC cell lines (5-8F, S26, and CNE2), while HEK293T cells were purchased from ATCC. All cell lines were grown in Dulbecco’s modified Eagle’s medium (DMEM, Gibco, USA) with 10% fetal bovine serum (FBS, Gibco, USA) at 37 °C in a humidified environment containing 5% carbon dioxide. Using a mycoplasma testing kit (Vazyme, China), we found that none of the cell lines were infected with the pathogen. Cell lines were maintained in a healthy physiological state by passaging them every two days when they reached approximately 80–90% confluency and were not passaged for more than two months.

### Western blotting and antibodies

With a phosphatase and protease inhibitor cocktail (Beyotime, China), cells were lysed and sonicated in RIPA (Beyotime, China) lysis solution. Proteins were separated by SDS‒PAGE and then transferred to a PVDF membrane (Merck Millipore, USA). Following 1 h at room temperature in a buffer containing 5% skim milk (BD Biosciences, USA) and 0.1% Tween 20 (Beyotime, China), the membranes were blocked. The membrane was then treated with the primary antibodies specified overnight at 4 °C, followed by incubation with goat anti-mouse or anti-rabbit secondary antibodies conjugated with horseradish peroxidase (HRP) (Cell Signaling Technology, USA) for 1 h. PierceTM ECL western blotting substrate (Thermo Fisher, USA) and Bio-Rad ChemiDoc Touch were used to detect and analyze the proteins. Antibodies against the following targets were used in this study: phospho-mTOR, mTOR, LC3B, P62, cleaved caspase 3, γ-H2A (5536, 2983, 3868, 23214, 9661, 2577, Cell Signaling Technology, USA), phospho-RPA2 (381220, Zen BioScience, China), actin (A5441, Sigma‒Aldrich, USA), RPA2 (ab76420, Abcam, USA), and Ki-67 (14-5698-82, Thermo Fisher, USA).

### Immunofluorescence staining

Glass coverslips were used to cultivate cells, which were then treated with HAMNO for the durations specified before being fixed in 4% paraformaldehyde for 20 min. Following a 10-min permeabilization in 0.3% Triton X-100 (Beyotime, China) in phosphate buffer saline (PBS), a 1-h blocking in 5% goat serum (Gibco, USA) and 0.3% Triton X-100 in PBS, and an overnight incubation at 4 °C with the appropriate primary antibodies, the cells were analyzed. Three washes in PBS were followed by incubation with a 1:750 dilution of an Alexa-conjugated secondary antibody after the cells had been stained. After 10 min in antifade mounting solution containing 4′,6-diamidino-2-phenylindole (DAPI, Beyotime, China), the cells were observed under a 63 × objective in a confocal laser scanning microscope (Carl Zeiss, Germany). The software package Zen 3.4 blue edition was used to calculate the relative fluorescence intensities of LC3B and γ-H2A. In each of three separate tests, ≥ 50 cells were counted from each group.

### Cell proliferation, colony formation and tumorsphere formation

Increased doses of HAMNO (NSC111847, InvivoChem, USA) were used to stimulate cell growth in CNE2, S26, and 5-8F cells plated at 1000 cells per well in 96-well plates. At the given period, cell viability was determined by counting viable cells after staining with Trypan blue (Gibco, USA). For analysis of cell confluence, plates were automatically monitored and recorded every 4 h by the IncuCyte S3 system (Essen BioScience, USA), and cell proliferation rates were evaluated by IncuCyte 2021C software. Colony formation assays were performed by seeding duplicate wells of 6-well plates with 1000 cells and then exposing them to X-ray doses of 0, 1, 2, 3, 4, and 5 Gy via a Rad Source R2000 irradiator. Cells were fixed with 4% paraformaldehyde for 10 min and then stained with 0.5% crystal violet solution after plate colony formation (12–14 days). With a light microscope, colonies with > 50 cells were tallied. The survival fraction was normalized to the number of colonies of nontreated cells. For 3D anchorage-free colony formation, 2000 S26 and 5-8F cells in DMEM enriched with 10% FBS were plated into 96-well ultralow attachment microplates (Corning, USA) and monitored by the IncuCyte S3 system. The culture medium was refreshed every three days throughout the experimental period. The volume of 3D colonies was calculated using the following formula: Volume = (4/3) πR^3^. For soft agar colony formation, cells were suspended in 100 μL of medium consisting of 10% FBS with 0.3% agar (Sigma‒Aldrich, USA) at 100 cells per well and seeded in 96-well plates with 0.6% bottom agar. The sizes of the colonies were determined by taking photographs of tumor spheres utilizing a phase-contrast microscope (Olympus microscope IX71, Japan). There were ≥ three separate experiments performed in triplicate.

### Immunohistochemistry (IHC)

IHC was performed according to the standard protocol. Antigen retrieval was carried out by boiling paraffin-embedded sections in EDTA (pH 8.0, 0.05% Tween 20, 1 mM EDTA) (Sigma‒Aldrich, USA) for 20 min. After being treated with primary antibodies against Ki-67 and cleaved caspase 3 diluted 1:200 in 3% bovine serum albumin (BSA) in PBS at 4 °C overnight, the sections were blocked with 3% goat serum. The Dako REAL EnVision Detection System (Dako, Denmark) was employed for immunostaining via HRP conjugates. Two expert pathologists utilized the immunoreactivity score (IRS) technique to quantify cleaved caspase 3 and Ki-67 expression. The proportion of tumor cells that were positive was measured and assigned a score between 1 (25%) and 4 (> 75%). Zero, one, two, and three indicated no, light yellow, moderate, and dark brown staining, respectively. The extent was multiplied by the intensity to obtain the final IHC score.

### Transmission electron microscopy

Ten-centimeter dishes were used for cultivating 5-8F or S26 cells, and then, they were treated with 10 μM HAMNO for 24 h. After being scraped gently with a cell scraper (Corning, USA), the samples were fixed in 2.5% glutaraldehyde (Sigma‒Aldrich, USA) for 5 min at room temperature after drug treatment. Following overnight fixation in 2.5% glutaraldehyde at 4 °C, the cells were centrifuged at a slower speed (3000 rpm min^−1^) to collect the precipitate. Electron microscopy (JEM-1400flash) was used to examine the samples at Sun Yat-sen University.

### Quantitative RT–PCR

The RNeasy Mini Kit (Qiagen, Germany) was used to extract total cellular RNA, and the QuantiTect Reverse Transcription Kit (Qiagen, Germany) was used to reverse-transcribe 1 μg of total RNA. Using the QuantStudio 7 Flex (Thermo Fisher, USA) and TB Green® Premix Ex Taq™ II (TaKaRa, Japan), we carried out real-time quantitative PCR. All PCRs were run in triplicate, and the 2 − ΔΔCq technique utilized for assessing the amplification products and relative expression levels were calculated via the housekeeping gene β-actin as a standard. Additional file 1: Fig. S3 contains the amplification primers for SQSTM1, WIP1, GABARAPL1, and β-actin.

### Glucose uptake assay, lactate production and intracellular ATP level measurement

After 2 h of incubation with 100 μg/mL 2-deoxy-2-[(7-nitro-2,1,3-benzoxadiazol-4-yl)amino]-d-glucose (2-NBDG, Cayman Chemical, USA) in glucose-free medium, fluorescence was measured via the IncuCyte S3 system at excitation and emission wavelengths of 485 nm and 535 nm. Following the manufacturer’s protocol, lactate was measured in cell lysates using the CheKine™ Micro Lactate Assay Kit (Abbkine, China). A total of 1 × 10^6^ cells were centrifuged at 4 °C after being lysed in 200 μL/well passive lysis buffer on ice for the ATP assay. The ATP content in the supernatant was determined using the Molecular Probes® ATP Determination Kit (Invitrogen, USA) in adherence to the recommended protocol. There were three sets of each experiment.

### In vivo* xenograft evaluation*

The Sixth Affiliated Hospital of Sun Yat-sen University’s Committee on the Ethics of Animal Studies examined and authorized all animal studies (IACUC-2022052701). All authors followed all applicable ethical guidelines for the use of animals in scientific studies. We ordered four-week-old female BALB/c nude mice from Beijing Laboratory Animal Co., Ltd. Mice were kept in microisolator cages with a 12-h light/12-h dark cycle, 18–22 °C temperatures, and 45% humidity. Subcutaneous injections of S26 cells (1 × 10^6^ cells in 100 μL of PBS) were made in the flanks of mice. Every three days after therapy, the tumor volume was determined using the following formula: volume = length × width^2^ × 0.5. When the xenograft tumor diameters reached ~ 5 mm, treatment was initiated. Animals were randomly grouped before receiving vehicle control, treatment, or combination therapy. HAMNO (2 mg/kg, 5% DMSO, 40% PEG300, 5% Tween 80) and DMSO as a vehicle control were administered intraperitoneally every 3 days twice. For combination therapy, mice were either nonirradiated or irradiated with 6 Gy once, followed by treatment with HAMNO (1 mg/kg) twice on day 2 and day 5 after irradiation. Injections of 60 mg/kg hydroxychloroquine (Sigma‒Aldrich, USA) were given intraperitoneally on days 1, 3, and 6. Mice were euthanized at the experimental end point, and xenografts were harvested, fixed in 10% formalin overnight, and paraffin embedded for histologic analysis.

### Flow cytometry apoptosis assay

S26 and 5-8F cells were collected 48 h following HAMNO, DMSO or irradiation therapy and subsequently subjected to an apoptosis assay. Apoptosis analyses were performed via an Annexin V-APC/7-AAD Apoptosis Detection Kit (Multi Sciences, China). Briefly, we centrifuged the samples at 300×*g* for 5 min to separate detached cells from the supernatant and after EDTA-free trypsin recovery. Annexin V incubation reagent (1% Annexin V-APC and 1 × 7-AAD solution) was used to incubate the samples at room temperature for 30 min in the dark after they were resuspended in 500 μL of 1 binding buffer. A cytoFLEX flow cytometer was used to measure the apoptosis frequency, and the data were processed using CytExpert 2.2. APC − /7-AAD − cells were considered viable cells, APC + /7-AAD − cells were considered early apoptotic cells, and APC + /7-AAD + cells were considered late apoptotic or dead cells. The mean and standard deviation were calculated for n = 3 independent biological replicates.

### Gene set enrichment analysis (GSEA) and gene set variation analysis (GSVA)

The GSE12452 dataset was downloaded from the Gene Expression Omnibus (GEO) database. High RPA1/RPA3 expression was defined as the top 50% of individuals in the GSE12452 dataset, while low RPA1/RPA3 expression was defined as the bottom 50% of patients. We assessed autophagy-associated genes and made a customized “autophagy‒lysosome signature” via a previously described method [13]. Then, we used GSEA using the R package clusterProfiler to find statistically significant functional variations between the two groups [14]. Pathways with a normalized enrichment score (|NES|) > 1) and a p value < 0.05 were considered significantly enriched. The GSVA score was calculated for each sample in the TCGA COAD based on the expression of genes in the gene set using the GSVA R package [15]. The GSVA scores of the two groups were compared using the Mann‒Whitney U test, and a p value < 0.05 was considered statistically significant. GSVA was utilized to assess the change and variation in pathway activities based on previously published gene signatures [16].

### Statistical analysis

Except when otherwise noted, all statistical testing was performed using GraphPad Prism version 8.3.0. Unless otherwise indicated, data that had a normal distribution are reported as the mean ± SD of triplicate experiments. Student’s unpaired t test was used to compare one single treatment to one control. Multiple treatment or condition studies were analyzed using one- or two-way ANOVA followed by a Dunnett or Tukey multiple comparison test to compare the means of each treatment to those of a predetermined control group. For the data that were not normally distributed, they were reported as the median with a 95% confidence interval (CI). Statistical significance was assessed using the following thresholds throughout the paper: *, p 0.05; **, p 0.01; ***, p 0.001. The primary raw data are available at www.researchdata.org.cn, which is the Research Data Deposit. This document includes its data sources.

## Results

### The RPAi HAMNO decreases nasopharyngeal carcinogenesis

**Fig. 1:**
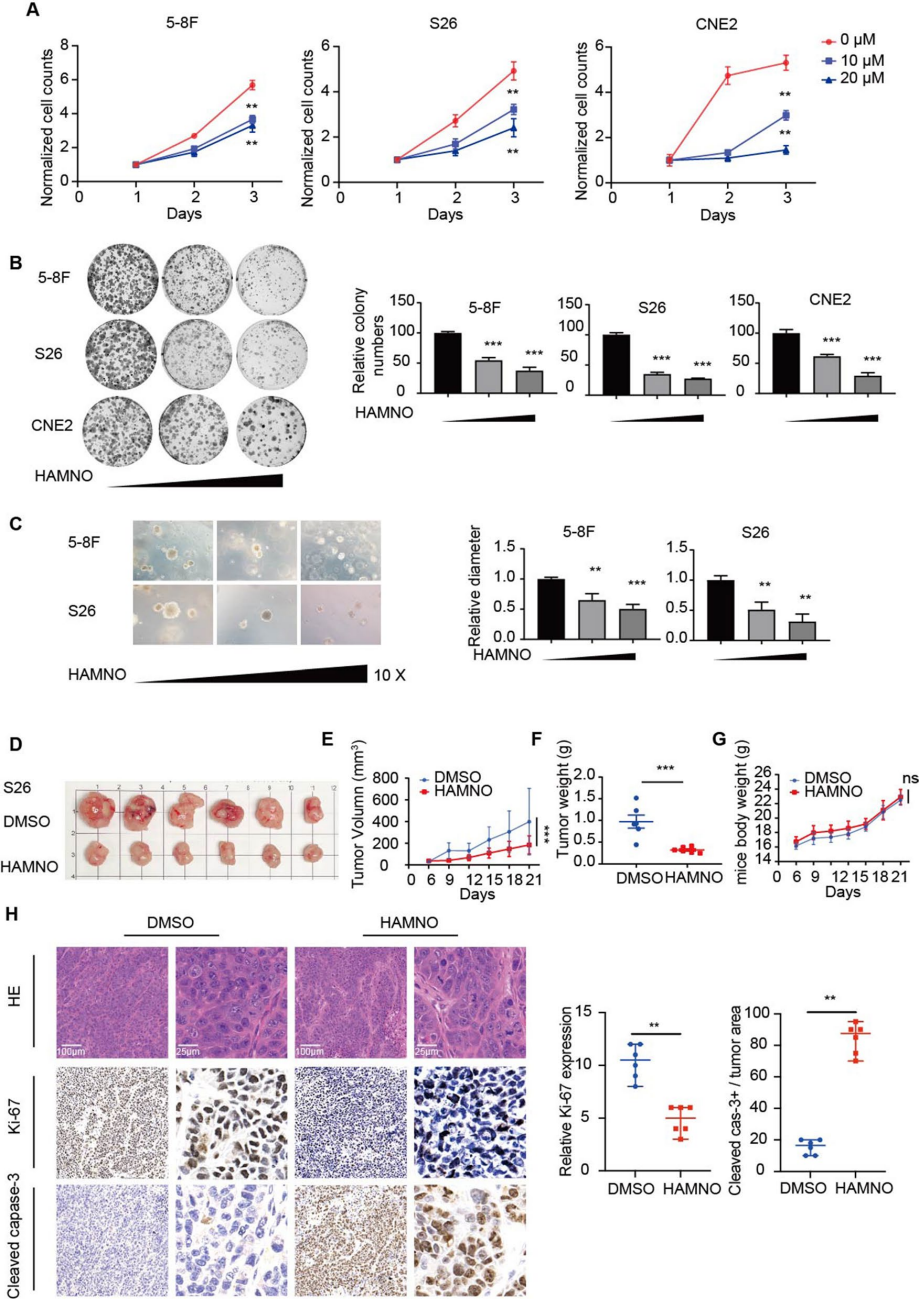
The RPAi HAMNO impairs the aggressive phenotype of NPC cells. **a** Cell growth curves for DMSO- and HAMNO-treated (10/20 μM) 5-8F, S26 and CNE2 cells at the indicated times. **b** Inhibition of RPA by HAMNO treatment impairs in vitro colony formation in DMSO- and HAMNO-treated (10/20 μM) NPC cell lines. Crystal violet staining of representative cells from three separate studies is shown (left). Focal adhesions were counted (on the right). **c** Representative images of tumorspheres and quantification of diameters from 5-8F and S26 cells. Soft agar colony formation assay of 5-8F and S26 cell lines treated with HAMNO (0, 10, 20 μM) for 12 days after seeding. Magnification, × 10. **d**–**g** The image shows the sizes of the tumors in nude mouse xenograft models at the end of the experiment D. Scale bar, 1 cm. Xenograft tumor growth curve E, weight F and mouse body weight G of S26 cells in nude mice treated with HAMNO at 2 mg/kg. Error bars in e and g mean ± SEM (n = 6 per group). ns = nonsignificant, two-way ANOVA with Dunnett’s multiple comparisons test. Data in f are shown as the mean ± SD (n = 6/group). Student’s t test. **h** Tumor slices from mice that were treated differently were stained with hematoxylin and eosin (HE) and IHC for Ki-67 and cleaved caspase 3 (left). Scale bar = 100 μm or 25 μm. The percentage of tumor cells that were positive for Ki-67 and cleaved caspase-3 in the various S26 xenograft tumor groups (right). The statistical analysis for H was performed using the Kolmogorov‒Smirnov test, and the data are presented as medians with 95% CI

When comparing untreated and treated NPC cells, we found that proliferation and colony formation were remarkably suppressed following therapy with the RPAi HAMNO (Fig. 1a, b). In addition, we employed a soft agar clonogenic assay by treating NPC cells with increasing doses of HAMNO and found that the tumorspheres of 5-8F and S26 cells were smaller in size overall, and the effect was dose-dependent (Fig. 1c). The findings above indicate that RPA controls the growth of NPC cells.

Drug treatment in the xenograft model led to a substantial antiproliferative effect on the tumor growth of S26 xenografts and no body weight loss (Fig. 1d–g). In contrast, no antiproliferative effect was noted in tumor-bearing mice treated with DMSO. IHC analysis of the xenograft tumors confirmed a significant reduction in the proliferation marker Ki-67 and an increase in the marker of apoptosis cleaved caspase-3 in HAMNO-treated cells (Fig. 1h), validating the antiproliferative effect of RPA inhibition in vivo. These findings confirm that RPA inhibition has a significant impact on reducing NPC cell growth both in vivo and in vitro.

### Pharmacological suppression of RPA enhances the radiosensitivity of NPC cells

**Fig. 2:**
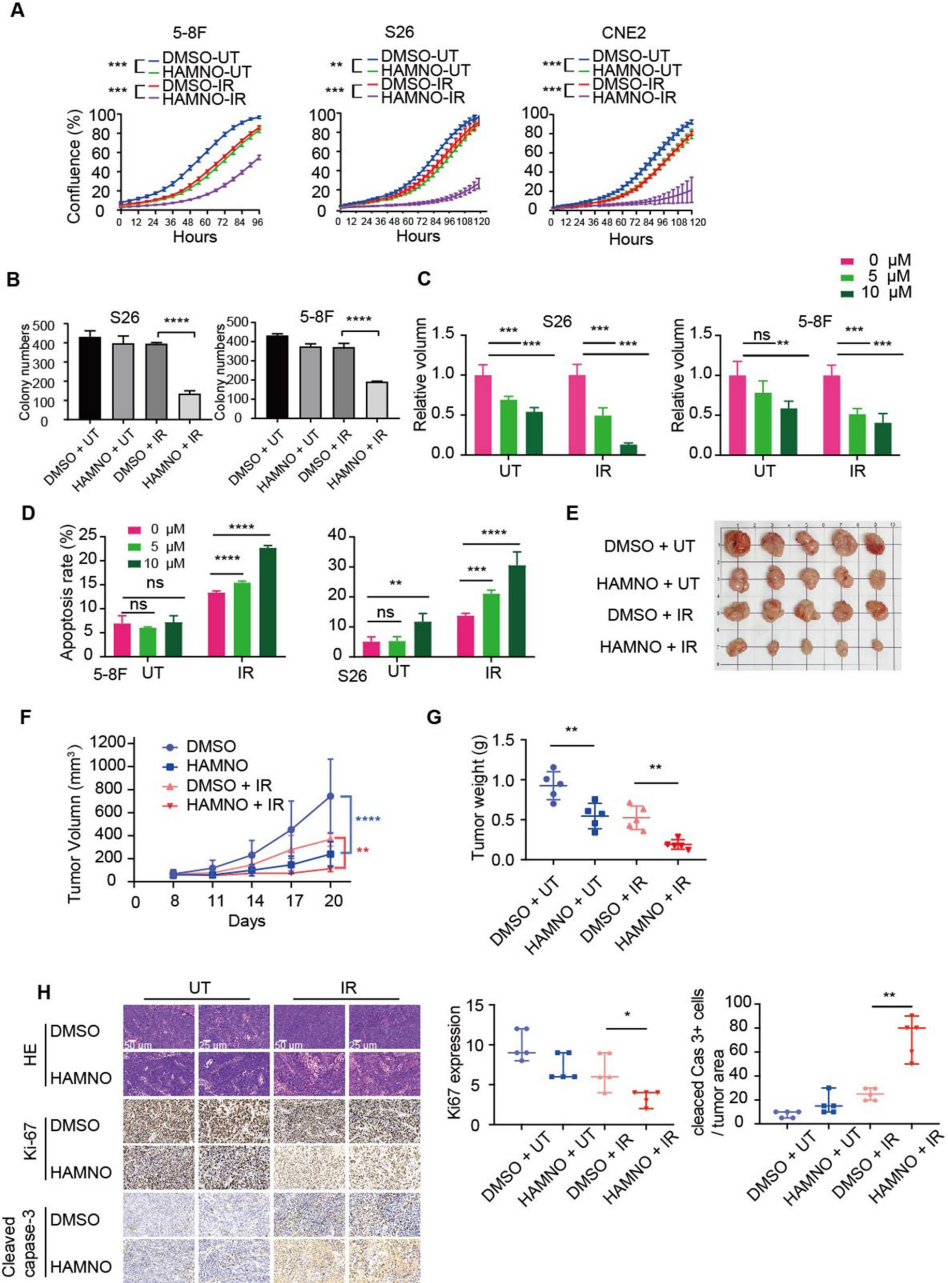
Inhibition of RPA enhances the antitumor effect of irradiation in NPC. **a** The proliferation of NPC cells with single or combination treatment was monitored with an IncuCyte system every 6 h after seeding. **b** Clonogenic growth assay of NPC cell lines treated for 12 days with the indicated treatment (HAMNO, 0, 5 and 10 μM). Cells were visualized using crystal violet staining. **c** Tumorsphere formation assay in NPC cells treated with a single inhibitor or combined with IR (left) following 10 days of culture. The cellular sphere volume was measured by ImageJ (right). **d** The percentage of apoptotic cells induced by RPAi (0, 5 and 10 μM) or IR alone or in combination for 48 h was determined via flow cytometry and Annexin V/7-AAD staining. The histogram represents the quantification of three independent experiments. The apoptosis rate was defined as the proportion of Annexin V-APC-positive and 7-AAD-negative cells out of the total number of cells. **e**–**g** Tumor formation in xenografted mice; representative tumor size images e of S26 cells in nude mice treated with HAMNO at 1 mg/kg, tumor volume over time (**f**) and average weight G of the excised tumors. **h** HE staining and IHC staining of Ki-67 and cleaved caspase 3 in tumor sections from mice with different treatments (left). Scale bar = 50 μm or 25 μm. Quantification of the proportion of Ki-67- and cleaved caspase-3-positive cells in each group of S26 xenograft tumors (right). The statistical analysis for H was performed using the Kolmogorov‒Smirnov test, and the data are presented as medians with 95% CI

Since RPA1 and RPA3 have been identified as potential radiotherapy targets for NPC [12, 17], we next sought to investigate whether pharmacologic inhibition of RPA exerts a radiosensitizing effect on NPC cells. To better observe the synergistic effect of RPAi and radiation, we chose low-dose HAMNO (5 µM) and irradiation (IR) doses for NPC cell treatment. Although HAMNO or IR treatment alone only moderately attenuated cell growth and colony formation, we observed that combined treatment greatly increased radiation sensitivity (Fig. 2a, b and Additional file 1: Fig. S1a). Similarly, tumorspheres for S26 and 5-8F cells treated with HAMNO or IR alone showed a modest response, while the combination treatment resulted in significant suppression of tumorsphere formation (Fig. 2c and Additional file 1: Fig. S1b). Together, according to these findings, IR alone was not as effective in decreasing NPC viability as targeting RPA in combination with IR. DNA damage upon IR is a major cause of the cell apoptosis response. To assess cell apoptosis, we estimated the proportion of apoptotic NPC cells after single or combination therapy using flow cytometry analysis with Annexin V-7-AAD labeling. The cell apoptosis rate was clearly increased in the combined HAMNO and IR treatment group compared to the DMSO control and HAMNO or IR alone groups of 5-8F and S26 cells (Fig. 2d and Additional file 1: Fig. S1c). As a result, the NPC cell in vitro radiosensitivity was increased by pharmacologic RPA inhibition. To test whether RPA inhibition increases the radiosensitivity of NPC cells in vivo, we created xenograft models to further verify our findings. Consistent with the in vitro results, we observed that combined therapy significantly inhibited S26 xenograft development compared to solo treatment (Fig. 2e–g). Consistent with the finding of reduced tumor size (Fig. 2e), the xenografts with the combined treatment showed a remarkable reduction in both tumor volume and weight compared with those with HAMNO or IR treatment alone (Fig. 2f, g). Moreover, we found dramatically decreased cell proliferation (Ki67+) and increased cell death (cleaved caspase 3+) in the combined treatment group mice (Fig. 2h), while xenograft tumors treated with HAMNO or IR alone showed a modest response. Collectively, these results show that the radiosensitivity of NPC cells is increased by the antiproliferative activity of pharmacologic RPA inhibition both in vitro and in vivo, suggesting its potential clinical application alongside radiotherapy in the management of cases with NPC.

### RPA inhibition promotes autophagy

**Fig. 3:**
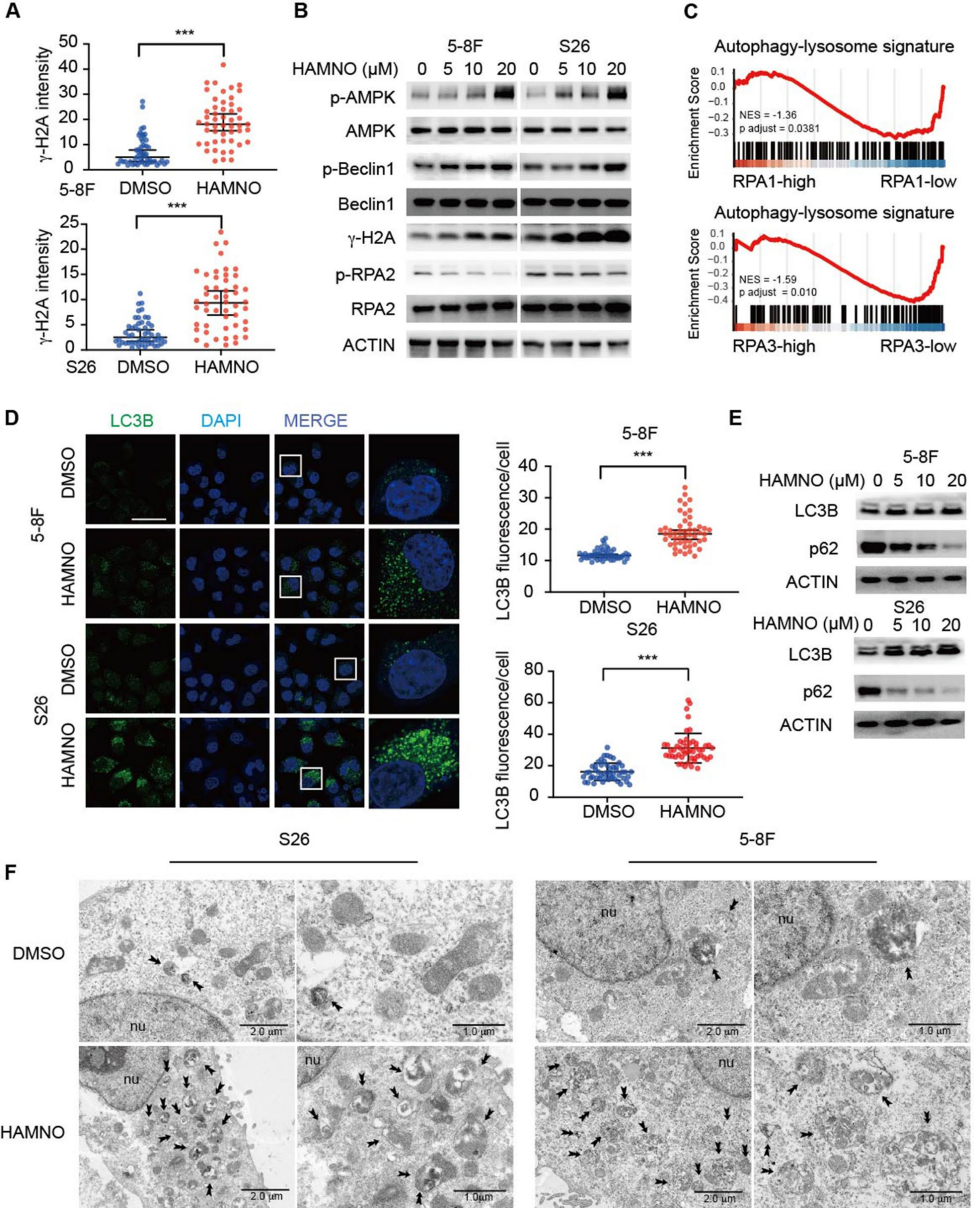
RPA inhibition in NPC cells enhances autophagic flux. **a** γH2A staining. Nuclear γH2A intensity was measured. The graph corresponds to the quantification of 50 cells for each group with three independent experiments. Blue, DAPI. Scale bar = 50 μm. **b** Immunoblot analyses of cell lysates were performed to determine the levels of p-AMPK, total AMPK, p-Beclin-1, total Beclin-1, γH2A, p-RPA2, total RPA2 and β-actin and are representative of three independent experiments. **c** GSEA identified the autophagy‒lysosome genes significantly elevated in the low RPA1/3 expression group in the GSE12452 gene expression data. **d** Quantification of LC3B staining after treatment with vehicle or 15 μM HANMO for 24 h. Blue, DAPI. Scale bar = 50 μm. **e** NPC cell lines were treated with DMSO or HAMNO at the indicated dose for 24 h to assess flux. Immunoblot analyses of cell lysates were performed to determine the levels of LC3B, p62 and β-actin. **f** Electron microscopy showed the ultrastructure of autolysosomes in 5-8F and S26 cells following 24 h of HAMNO treatment. Scale bar = 1 or 2 μm as indicated. In (**a**, **b**) and (**d**–**f**), all experiments were performed with three biological replicates, and for immunoblots, a representative image is shown. The statistical analysis for (**a**) and (**d**) was performed using the Kolmogorov‒Smirnov test, and the data are presented as medians with 95% CI

Given that RPA inhibition leads to a remarkable antiproliferative effect upon IR, we assessed the biological consequences of RPA inhibition. The DDR, which is a result of IR, was next evaluated in the cell lines via an examination of histone H2AX (γH2AX) phosphorylation, an indicator of double-strand DNA breaks (DSBs). We observed severe DNA damage upon RPA inhibition (Fig. 3a, b and Additional file 1: Fig. S1d). We discovered that RPAi therapy increased the levels of phosphorylated and active Beclin-1 (an autophagic substrate) and AMPK⍺ (T172) (Fig. 3b). Notably, AMPK and Beclin1 have emerged as critical constituents in the initiation of autophagy and the orchestration of autophagosome assembly [1, 2]. Guided by these insights, we postulated that the inhibition of RPA could potentially trigger enhanced autophagic signaling even before the physical formation of autophagosomes within the context of NPCs. We discovered, via GSEA of the GSE12452 dataset, that NPC samples exhibiting reduced RPA1/RPA3 expression were considerably more enriched in gene sets linked to the autophagy‒lysosome pathway than those with high RPA1/RPA3 expression (Fig. 3c). To further determine whether RPAi stimulates autophagy, we performed immunofluorescence and western blot analyses to assess the formation of LC3B puncta, a marker of autophagosomes or autolysosomes, and the conversion of LC3B-II in NPC cell lines. The NPC cells treated with HAMNO showed that LC3B puncta were significantly increased and that the conversion of LC3B-II was enhanced (Fig. 3d, e). Additionally, RPA downregulation reduced the stability of p62, a dynamic indicator of the onset of autophagic flux, reinforcing the idea that RPA downregulation increases autophagic flux (Fig. 3e). Compared to DMSO controls, HAMNO-treated NPC cells had a greater total number of autophagic vesicles due to the presence of more autolysosomes, as shown via morphometric and electron microscopy studies (Fig. 3f). Altogether, these findings suggest that RPA inhibition stimulates autophagy and may have implications for therapeutic approaches in the treatment of NPC.

### RPA suppression is involved in metabolic activity deregulation

**Fig. 4:**
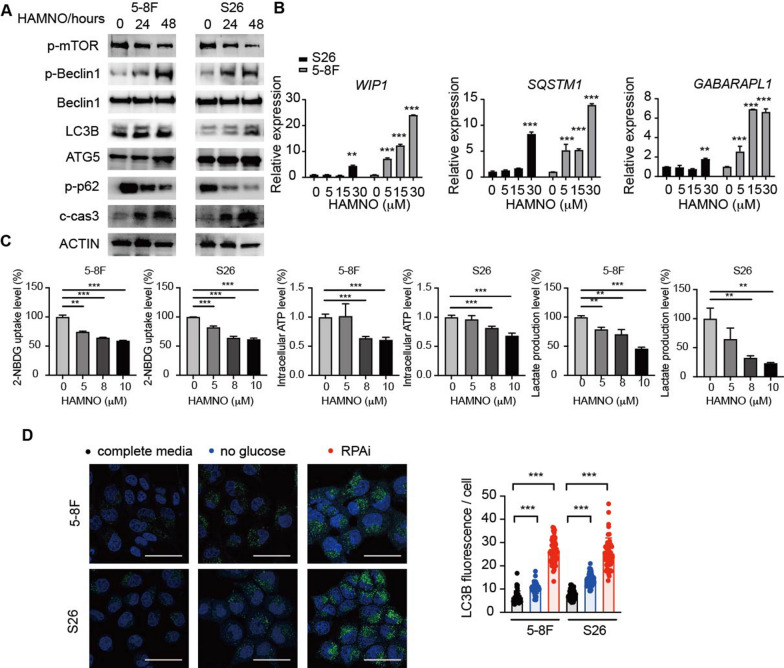
Pharmacological inhibition of RPA disrupts metabolic activities. **a** S26 and 5-8F cells were subjected to 24- or 48-h treatment with DMSO or 10 μM HAMNO, followed by immunoblot analysis to assess pmTOR, pBeclin1, total Beclin1, LC3B, ATG5, p62, cleaved caspase 3 and ACTIN levels. **b** S26 and 5-8F cells were treated with increasing doses of HAMNO (concentrations in μM shown) for 24 h. Relative gene expression of *WIP1*, *SQSTM1* and *GABARAPL1* was analyzed by qRT-qPCR. mRNA levels were normalized to *ACTB* mRNA. Relative expression quantified via the double delta Ct method is plotted. Data are representative of three biological replicates. **c** Effects of RPAi treatment at the indicated dose for 24 h on glucose utilization, lactic acid production, and intracellular ATP levels in 5-8F and S26 cells. Data are representative of five biological replicates. d S26 and 5-8F cells were treated with HAMNO or cultured in medium containing dialyzed FBS and no glucose (16 h). Quantification of LC3B staining is presented for three biological replicates

The molecular origin of the RPAi-induced increase in autophagic flux was next examined. AMPK activation, which activates autophagy by inhibiting mTORC1, is an initial process that induces autophagy and metabolic activity. RPA inhibition increased AMPK activity while decreasing mTOR signaling (Figs. 3b, 4a and Additional file 1: Fig. S2a), indicating that RPA modulated AMPK-mTOR signaling-mediated autophagy. We further performed RNA-seq analysis on S26 and 5-8F cell lines treated with the RPAi within 48 h. Despite being nonsignificant, GSVA revealed that RPA inhibition enhanced the proliferation of autophagy-related genes (Additional file 1: Fig. S2b). We subsequently used quantitative PCR to confirm dose-dependent elevations in autophagy-related gene transcripts, particularly those encoding autophagy cargo receptors, following RPAi therapy. Consistent with the increased level of autophagy-related genes identified by GSVA, we observed markedly increased mRNA levels of WIPI1, GABARAPL1 and SQSTM1 (Fig. 4b). Thus, RPA inhibition enhances autophagy at the gene transcription and autophagic signaling levels.

In addition to the increase in autophagy signaling transcripts, the utilized RNA-seq investigations demonstrated that RPA inhibition inhibited the transcription of glycolysis-related genes (Additional file 1: Fig. S2b). We reasoned that the decrease in glycolytic activity caused by RPA inhibition may contribute to the RPAi-mediated increase in autophagic flow that we noted. In accordance with the regulatory role of AMPK in energy sensing, we extended the examination and found that RPA suppression decreased glucose uptake, lactic acid production and intracellular ATP levels (Fig. 4c). We hypothesized that the reduction in glycolytic activity induced by RPA inhibition may be responsible for the observed increase in autophagic flux. To support this, we performed an experiment by removing glucose from the growth medium of NPC cells. We observed an increase in autophagic flux upon glucose withdrawal, although the extent of the increase was less pronounced compared to the effect observed with RPA inhibition (Fig. 4d). As a result, we infer that RPAi therapy promotes autophagic flow both directly by changing signaling systems and indirectly by lowering glycolytic activity.

### The combination of RPAi and autophagy inhibition synergistically enhanced the radiotherapy effect of NPC cells

**Fig. 5:**
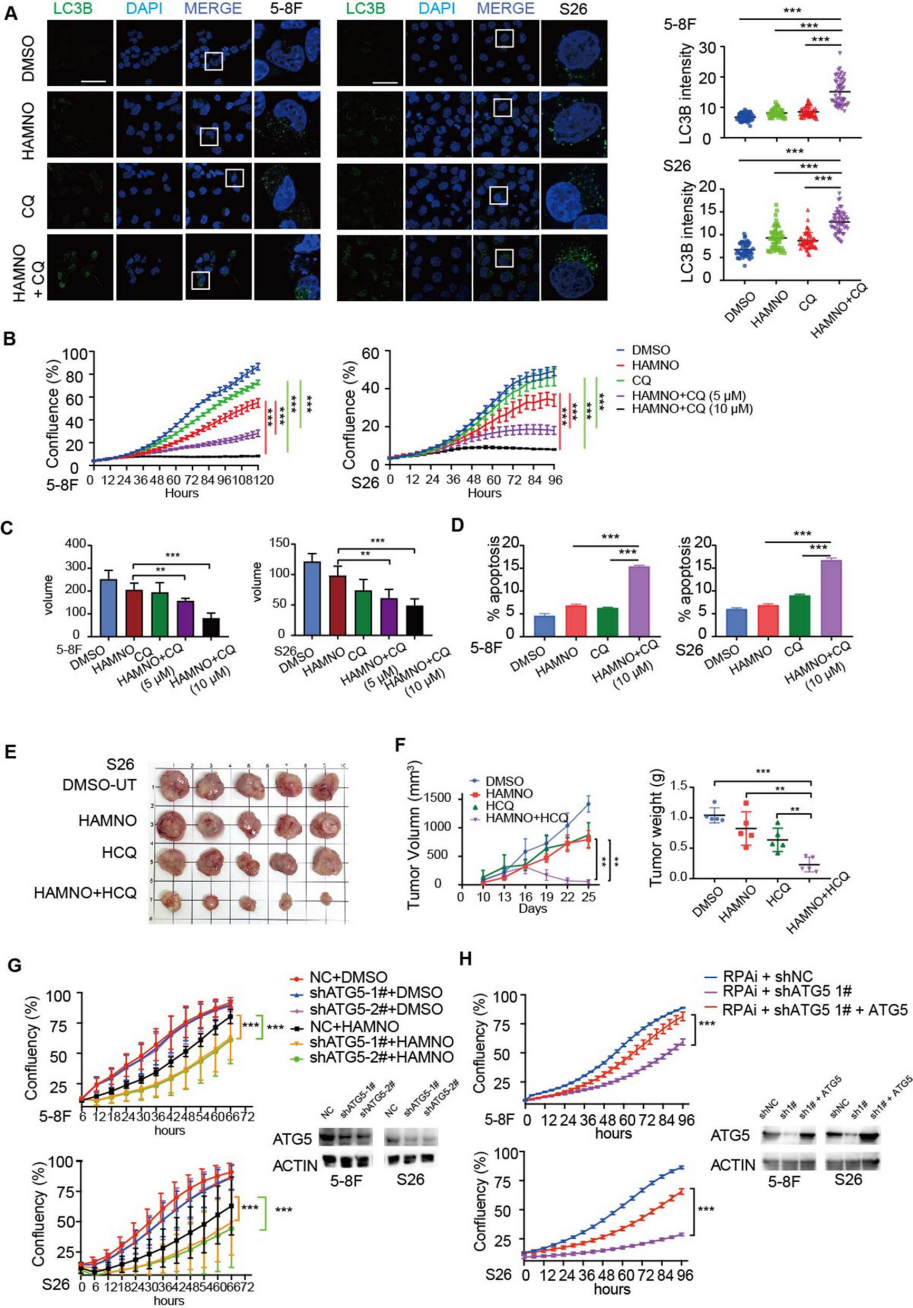
RPA inhibition enhanced the sensitivity of NPCs to hydroxychloroquine-mediated inhibition of autophagy. **a** Quantitative result of LC3B puncta in 5-8F and S26 cells following treatment with HAMNO (5 µM) in the absence or presence of CQ (10 µM) for 48 h. Scale bar = 50 μm. **b** Confluence of 5-8F and S26 cells treated with HAMNO (5 µM) alone or in combination with CQ (5 µM or 10 µM) was analyzed by the IncuCyte system. **c** Tumoroids treated with HAMNO (5 µM) alone or in combination with CQ (5 µM or 10 µM) were analyzed by the IncuCyte system. The volume was measured by ImageJ. The mean of five independent experiments is plotted. **d** The percentage of apoptotic cells induced by HAMNO or CQ alone or in combination for 48 h was determined via Annexin V/7-AAD staining and flow cytometry. The histogram represents the quantification of three independent experiments. Data are the mean of three independent experiments. **e** Images display tumor size at the end of treatment. **f** Xenograft tumor growth curve of S26 cells treated with HAMNO (1 mg/kg) or HCQ (60 mg/kg) alone or in combination (left). The scatter plot displays the tumor weight at the end of treatment (right). Error bars indicate the mean ± SEM. for n = 5 biologically independent animals. **g** Growth curves of ATG5 knockdown NPC cells treated with RPAi. **h** Cell growth curve of shATG5 knockdown-NPC cells transfected with pcDNA3.0-ATG5 overexpression plasmid or an empty vector control

We next examined whether RPA’s participation in NPC is reliant on autophagy and whether inhibiting both RPA and autophagy at the same time might reduce NPC cell development. As a result, we discovered that concomitant administration of RPAi and CQ, a lysosome acidification inhibitor and therefore an indirect regulator of autophagic flux, promoted autophagy (Fig. 5a). Concurrent treatment with RPAi and CQ synergistically suppressed the growth of NPC cells (Fig. 5b). Cells grew in 3D anchorage-independent spheroid culture conditions and were synergistic (Fig. 5c and Additional file 1: Fig. S2c). Additionally, low-dose RPAi therapy alone resulted in a minimal amount of apoptosis, while the combined treatment dramatically enhanced the apoptosis rate (Fig. 5d and Additional file 1: Fig. S2d).

We created xenograft models to determine whether the synergistic effects of NPC cells extend to in vivo tumor development. The combination treatment group showed tumor stabilization and partial regressions in terms of the excised tumors’ weight, volume, and size, suggesting tumor reduction when compared to that of the hydroxychloroquine (HCQ) or the RPAi monotherapy groups (Fig. 5e, f). Furthermore, in the concurrent therapy group, Ki67 expression rose while cleaved caspase 3 protein levels dropped (Additional file 1: Fig. S2e), indicating that proliferation and cell death were significantly inhibited in RPAi-treated NPC cells and more strongly inhibited after combination with an autophagy inhibitor. Furtheremore, we employed shRNA to knock down a key autophagy-related gene (*ATG5*), which is important in autophagosome membrane elongation and vesicle production. We therefore investigated whether pharmacologic inhibition of certain autophagic components would potentially synergize with RPA inhibition. As expected, we observed that the antiproliferative effects of HAMNO in NPC cell lines were strongly enhanced in *ATG5* knockdown cells (Fig. 5g). Additionally, the partial restoration of HAMNO’s inhibitory impact upon ATG5 overexpression (Fig. 5h) further underscores the intricate interaction between RPA inhibition and autophagic modulation.

**Fig. 6:**
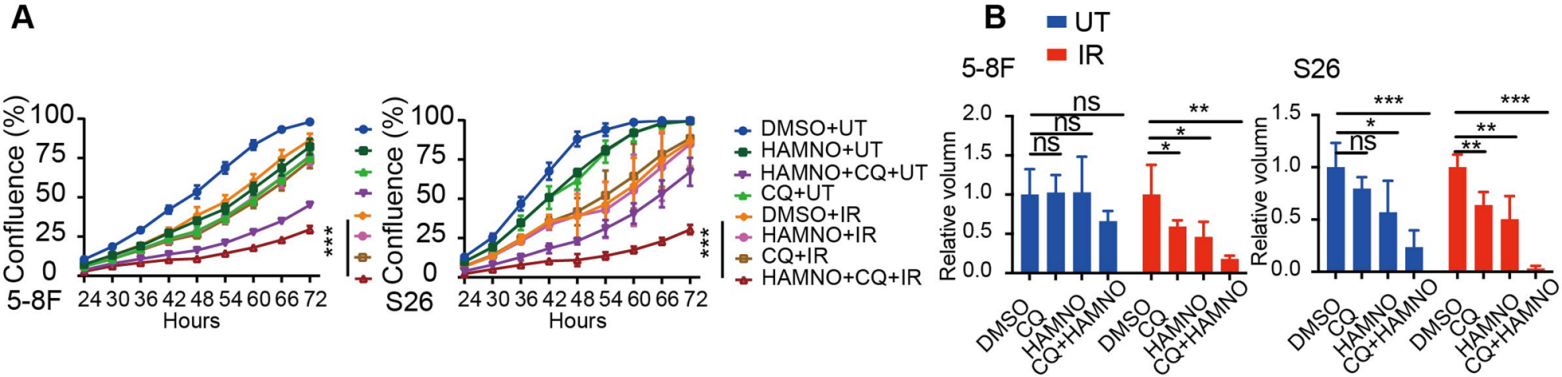
Concurrent inhibition of RPA and autophagy sensitized NPC cells to radiation. **a** Growth curves and **b** tumoroids of 5-8F and S26 cells treated with HAMNO (3 µM), CQ (5 µM) or IR (0.5 Gy), alone or in combination as indicated. The mean of three independent experiments is plotted

We anticipated that combined pharmacologic suppression of RPA and IR therapy would also synergize with autophagy inhibition after demonstrating a strong synergistic relationship between autophagy and RPA inhibition. We treated NPC cell lines with RPAi in conjunction with HCQ and IR and found a synergistic improvement in RPAi-induced growth inhibition in addition to a considerably greater apoptosis rate in combination-treated cells (Fig. 6a, b and Additional file 1: Fig. S2g).

## Discussion

Genomic instability is a well-known cancer hallmark, and developing drugs targeting the DDR is a potential therapeutic strategy for various solid cancers. Chemotherapeutic agents and therapeutic radiation initiate DNA damage and induce cell cycle arrest or apoptosis of tumor cells, which consequently leads to treatment resistance [18]. Therefore, agents that suppress DDR signaling pathways are supposed to potentiate the cytotoxic impact of chemotherapy and radiotherapy as ‘sensitizing agents’ and thus overcome resistance. The key component of DDR, ATR-CHK1, might be pharmacologically inhibited as a therapeutic strategy for cancer [19]. Clinical studies of certain ATR and CHK1 inhibitors demonstrated significant cytotoxicity, and numerous candidate molecules with enhanced safety profiles are now being explored (e.g., NCT03682289, NCT05071209, NCT02203513). However, the occurrence of off-target effects caused by ATR and CHK1 inhibition could involve pathways other than the DDR and cause cell death in both normal and cancer cells [11]. Furthermore, the embryonic mortality of mice lacking ATR and CHK1 suggested that directly blocking the ATR and CHK1 pathways might have deleterious effects. Thus, ATR-CHK1 pathway downstream components could be useful for treating cancer [11]. Following DSBs or at stalled replication forks, RPA binds to single-strand DNA (ssDNA) and therefore activates the ATR/CHK1 pathways, resulting in activation of the G2/M and intra-S phase cell cycle checkpoints and initiation of DNA repair. The RPA heterotrimers are downstream of the ATR substrate and may be a considerable pharmacological target for cancer therapy [20].

Because replication stress is higher in tumor cells and the leading cause of genome instability, targeting replication stress has enabled the discovery of new cancer vulnerabilities [21]. The RPA-ssDNA platform is a key sensor that triggers the DDR in reaction to genotoxic stressors, making it vital for genome integrity [22, 23]. HAMNO is a small molecule inhibitor of RPA that blocks checkpoint engagement in reaction to replication stress by inhibiting the link between RPA1 and ATR/ATRIP [10, 11]. This molecule works effectively with etoposide to induce replication stress and selectively increase cell death in cancer cells that exhibit constant DNA replication stress. This strategy has benefits in the clinic, as synergism of RPAis and other chemical drugs would enhance therapeutic efficacy while minimizing undesirable side effects [11]. We previously determined that RPA1 increases tumor growth and resistance to therapeutics in NPC, which affects the prognosis for individuals with NPC, using bioinformatic investigations and biological evidence [12]. Inhibition of RPA by HAMNO was shown to have a significant antitumoral impact on NPC cells both in vivo and in vitro. The standard therapeutic approach for NPC is radiotherapy. Nevertheless, radioresistance remains the major factor in suboptimal therapeutic outcomes and poor prognosis. Our findings showed that pharmacological inhibition of RPA increased the radiation-induced DDR and enhanced the radiosensitivity of NPC cells. These findings suggest that HAMNO may be useful in NPC treatment.

Autophagy functions as a double-edged sword in the mechanisms of radioresistance in NPC. While certain factors, such as lncRNA CASC19 and LACTB2, promote radioresistance by inducing autophagy, showing a positive correlation [24, 25], LUC7L2 conversely enhances resistance by inhibiting autophagic flux [26]. Our research demonstrated that inhibiting RPA increased autophagic flux, rendering NPC cells more responsive to autophagy inhibition. Moreover, radiation promotes autophagy in radioresistant NPC cells, and in turn, inhibition of autophagy reverses radioresistance [27]. Consistent with these findings, our study delved into the role of RPA in NPC radiotherapy. Notably, our investigation revealed that the concurrent inhibition of RPA and autophagy heightened the radiosensitivity of NPC cells, suggesting a promising avenue to enhance therapeutic efficacy. This novel mechanistic insight provides a foundation for the development of combination therapeutic strategies targeting both RPA and autophagy to overcome radioresistance specifically in NPC.

Additionally, our data demonstrated a notable escalation in DNA damage following RPA inhibition, suggesting a compelling relationship between DNA damage and RPAi-induced autophagy. DNA damage is a potential stimulus for autophagic initiation—an adaptive mechanism intended to mitigate cellular harm and restore homeostasis [28, 29]. Thus, one of the reasons for the heightened autophagic flux observed upon RPA inhibition might be a cellular strategy to counteract the stress induced by DNA damage.

Our investigations revealed that RPA inhibition induced a notable reduction in glycolytic activity. This observation raises intriguing questions about the potential links among RPA, metabolic pathways, and autophagic flux. We also found that RPAi activated AMPK and inhibited the mTOR signaling pathway. Given AMPK’s role as a key sensor in glycolysis and energy homeostasis [30], the activation of AMPK upon RPA inhibition not only aligns with autophagic initiation but also supports the idea that RPA inhibition might trigger a metabolic shift away from glycolysis. Consequently, we hypothesize that the enhanced autophagic flux resulting from RPA inhibition is, in part, attributed to the attenuation of glycolysis. The suppression of glycolytic activity potentially curtails the cell’s energy supply and prompts AMPK-driven autophagy as an alternative energy source. This intricate relationship among RPA inhibition, metabolic shifts, and autophagy further underscores the complexity of cellular responses under RPA modulation. While our observations hint at an intriguing interdependence between DNA damage and RPAi-induced multiple metabolic pathways, further targeted experiments are necessary to comprehend their underlying interactions.

Our study has several noteworthy strengths. Foremost, we elucidated the mechanisms underlying RPA inhibition-induced autophagy in NPC cells, offering new insights into autophagic regulation specific to NPC. The induction of autophagic flux, activation of the AMPK/mTOR pathway, and upregulation of autophagy-related genes were identified as key factors in this process. Second, our multifaceted investigation unveiled not only the intricate interplay among RPA inhibition, the DDR, and autophagy but also the involvement of cellular metabolic pathways. This revelation underscores the depth of cellular responses orchestrated under RPA modulation. Moreover, the investigation of combining autophagic inhibition (using CQ or genetic inhibition of ATG5) with RPA inhibition showed that this combination therapy is more effective in enhancing NPC’s antitumor response to radiation than monotherapy. This finding underscores the potential synergistic effects of a combination approach targeting multiple pathways.

We also acknowledge certain limitations in our study. Our study primarily assessed the short-term efficacy and therapeutic outcomes of the proposed treatment approach. Future research is warranted to investigate the long-term results, such as treatment durability, potential adverse reactions, and toxicity. Additionally, future studies exploring the direct links among RPA inhibition, glycolytic modulation, and autophagic induction are required for a comprehensive understanding of the intricate crosstalk within the cellular machinery.

## Conclusions

Our study has revelated the potential of RPA inhibition in the treatment of NPC. We demonstrated that pharmacological inhibition of RPA with HAMNO exhibited potent antitumor effects both in vitro and in vivo and enhanced the NPC response to radiotherapy.

We further elucidated the underlying mechanisms of RPA inhibition. We observed that inhibiting RPA activates the AMPK/mTOR signaling pathway, resulting in the induction of autophagic flux, which was supported by an increase in the expression of autophagy-related genes. Additionally, our research revealed a dual impact of RPA inhibition on cell metabolism. This molecule impairs glycolysis while simultaneously stimulating autophagy, creating a greater reliance on this cellular recycling mechanism.

Our findings lead us to propose a promising treatment approach for NPC. By utilizing RPA complex inhibitors to disrupt metabolic processes, particularly glycolysis, we induce a higher reliance on autophagy for the survival of NPC cells. This increased dependence on autophagy makes the cells more susceptible to the effects of autophagy inhibitors, such as CQ/hydroxychloroquine. Therefore, this combination therapy has the potential to enhance the effectiveness of radiotherapy through the synergistic effects of RPA inhibition and autophagy inhibition.

However, further research is required to validate the efficacy and safety of RPA inhibitors in a clinical setting. Although our study successfully revealed the mechanisms of RPA inhibition, a comprehensive understanding of the intricate molecular interactions involved remains imperative. Our future focus will be on revealing the precise mechanisms involved and identifying potential therapeutic targets.

Overall, our research highlights the therapeutic value of targeting RPA and metabolic pathways in NPC, providing a promising avenue for improving treatment outcomes and potentially overcoming the challenging treatment resistance of NPC to radiotherapy.

### Supplementary Information

**Additional file 1**. Representative Visuals Depicting Colony Formation Analysis, Tumor Sphere Formation Assay, IHC and the Percentage of Cells Undergoing Apoptosis.

## Data Availability

The data that support the findings of this study are available from the corresponding author upon reasonable request.

## Abbreviations

NPC: Nasopharyngeal carcinoma
RPA: Replication protein A
RPAi: RPA inhibitor
HAMNO: (1Z)-1-[(2-hydroxyanilino) methylidene] naphthalen-2-one
CQ: Chloroquine
AMPK: Adenosine 5′-monophosphate (AMP)-activated protein kinase
mTOR: Mammalian target of rapamycin
DNA: Deoxyribonucleic acid
DDR: DNA damage response
SD: Standard deviation
BSA: Bovine serum albumin
PBS: Phosphate buffer saline
HRP: Horseradish peroxidase
DAPI: 4′,6-Diamidino-2-phenylindole
IHC: Immunohistochemistry
2-NBDG: 2-Deoxy-2-[(7- nitro-2,1,3-benzoxadiazol-4-yl)amino]-D-glucose
IR: Irradiation
γH2AX: Phosphorylation of histone H2AX
DSBs: Double-strand DNA breaks
GSEA: Gene set enrichment analysis
GSVA: Gene set variation analysis
HCQ: Hydroxychloroquine
ssDNA: Single-strand DNA
HE: Hematoxylin and eosin staining
