# Supplementary material for: Targeting RPA promotes autophagic flux and the antitumor response to radiation in nasopharyngeal carcinoma
Source: J Transl Med. 2023 Oct 19;21:738. doi: 10.1186/s12967-023-04574-w (PMC10585929; doi:10.1186/s12967-023-04574-w)
Supplement: Supplementary file 1 — Additional file 1. Representative Visuals Depicting Colony Formation Analysis, Tumor Sphere Formation Assay, IHC and the Percentage of Cells Undergoing Apoptosis. [file 12967_2023_4574_MOESM1_ESM.pdf]

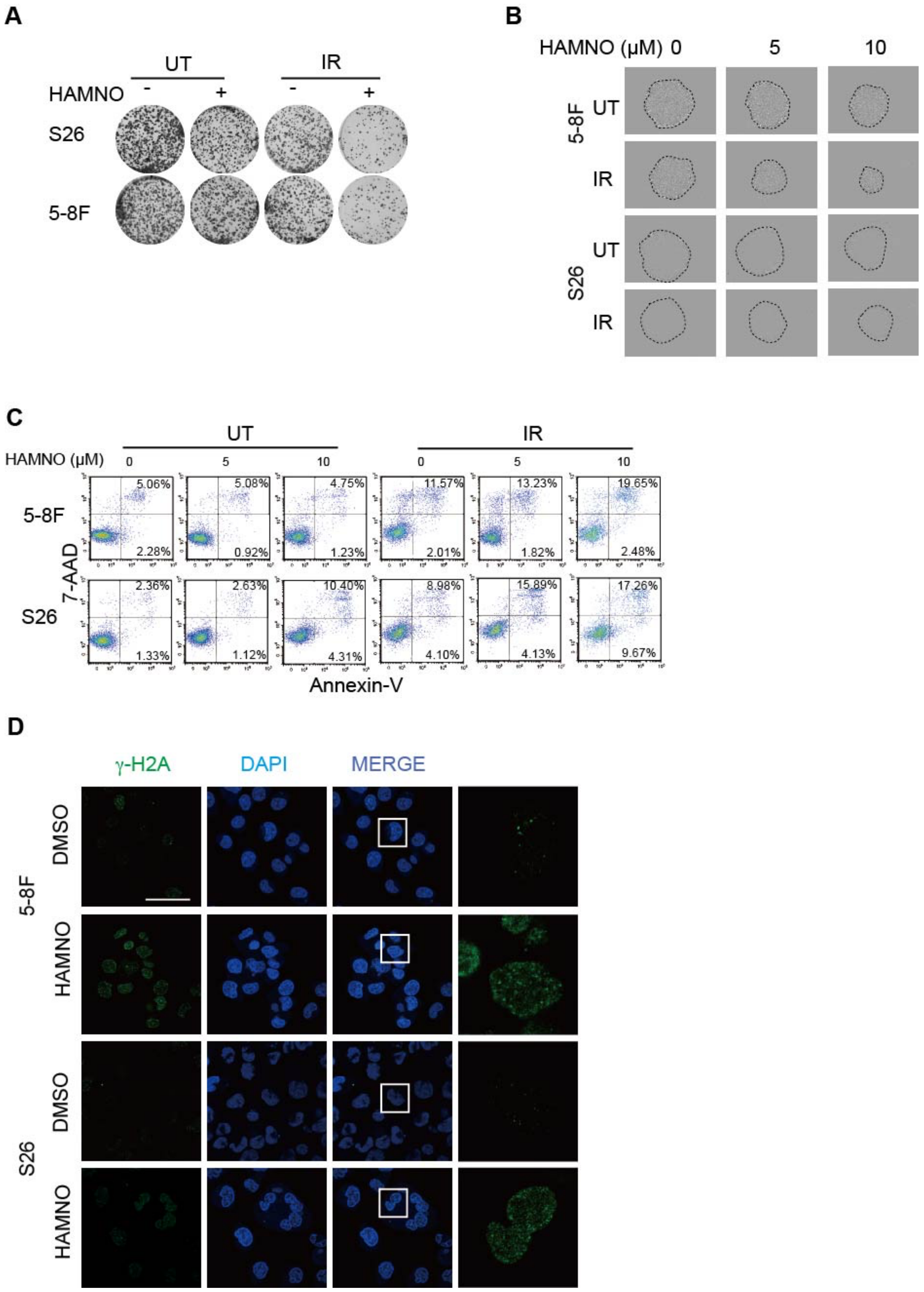

Additional file 1: Fig. S1

a Colony formation analysis of NPC cell lines treated with HAMNO, IR, or their combination. **b** Representative image of the tumor sphere formation assay in NPC cells treated with a single inhibitor or combination with IR after 12 days of cell

culture. Scale bar = 25/50 μm. **c** Percentage of cells in apoptosis induced by RPAi or IR alone or in combination for 48 hours was determined via Annexin V/7-AAD staining and flow cytometry. **d** Representative images of γH2A staining. Scale bar = 50 μm.

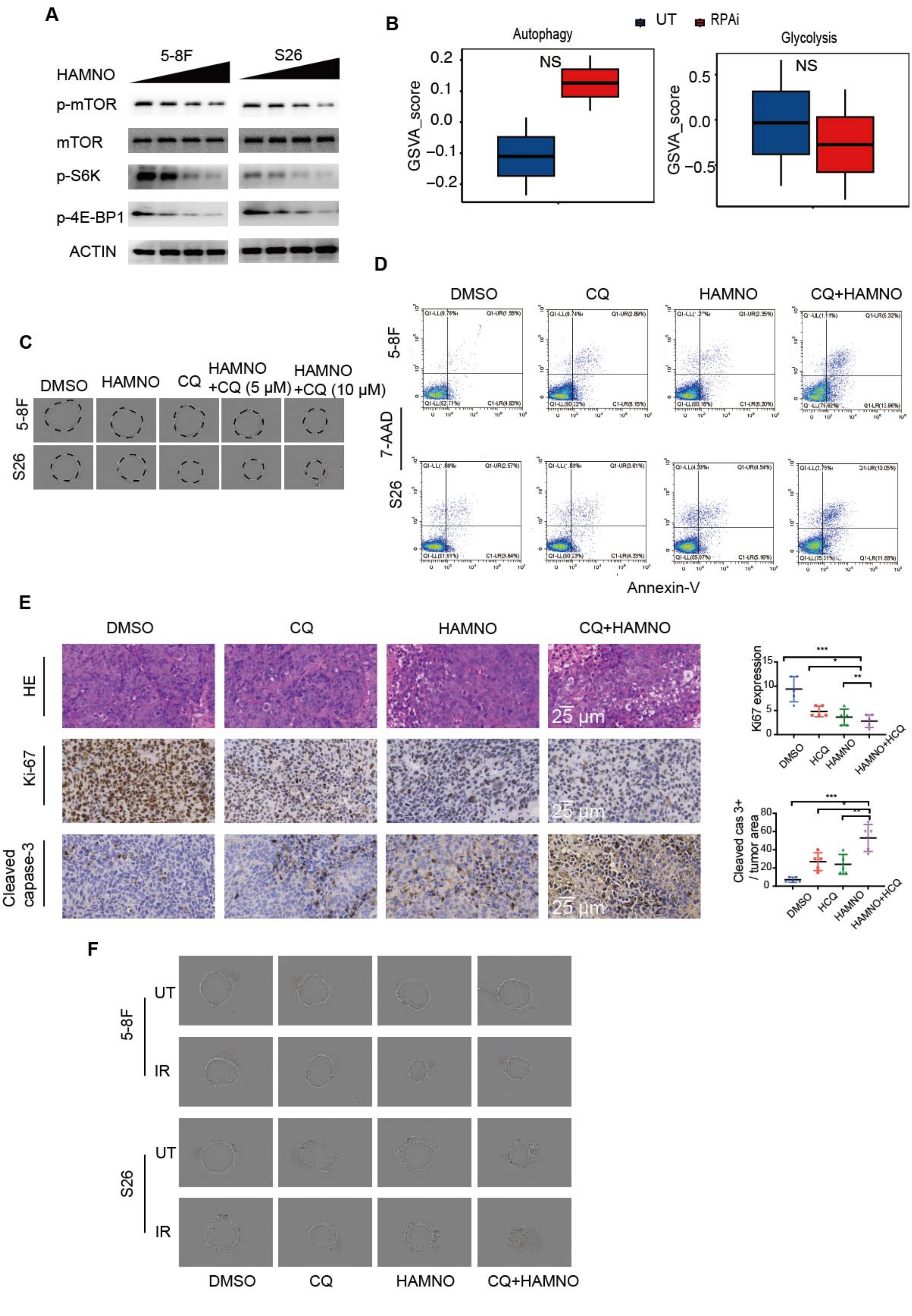

Additional file 1: Fig. S2

a 5-8F and S26 cells were treated with DMSO or HAMNO (10 μM, 20 μM or 30 μM) for 48 hours. Immunoblot analyses of cell lysates were performed to determine the levels of pmTOR, total mTOR, pS6K, p-4E-BP1 and ACTIN. **b** 5-8F and S26 were

treated with HAMNO (15 μM) for 48 h. Cells were collected and subjected to RNA-seq analysis. GSVA was completed to estimate the variation of different metabolic pathway activities in a nonsupervised manner. Each of the two cell lines was treated as a biological replicate. The GSVA scores for a gene set composed of autophagy-related and glycolysis genes were presented. GSVA scores of the two groups were compared using the Mann-Whitney U test. **c** Tumoroids treated with RPAi (5 μM) or CQ or IR alone or in combination were captured by the IncuCyte system. **d** Percentage of cells in apoptosis induced by RPAi or CQ alone or in combination for 48 hours was determined via flow cytometry and Annexin V/7-AAD staining. **e** Representative images of HE and IHC with indicated antibodies in tumor tissues from S26 models (left). Scale bar = 25 μm. IHC analysis of Ki67 and cleaved caspase-3 in tumor sections (right). **f** Tumoroids treated with HAMNO (5 μM), CQ or IR alone or in combination were captured by the IncuCyte system.

Additional file 1: Fig. S3

primers

QP-hGABARAPL1-Forward	**TTGTAGAGAAGGCTCCAAAAGCC**
**QP-hGABARAPL1-Reverse**	GGTCTCAGGTGGATTCTCTTCC
**QP-hWIPI1- Forward**	CTTCAAGCTGGAACAGGTCACC
**QP-hWIPI1- Reverse**	CGGAGAAGTTCAAGCGTGCAGT
**QP-hSQSTM11- Forward**	TGTGTAGCGTCTGCGAGGGAAA
**QP-hSQSTM1- Reverse**	AGTGTCCGTGTTTCACCTTCCG
